# Patterns of mental health problems and well-being in children with disabilities in Sweden: A cross-sectional survey and cluster analysis

**DOI:** 10.1371/journal.pone.0288815

**Published:** 2023-07-18

**Authors:** Torun Täljedal, Mats Granlund, Lena Almqvist, Fatumo Osman, Eva Norén Selinus, Karin Fängström

**Affiliations:** 1 Region Västmanland – Uppsala University, Centre for Clinical Research, Västmanland Hospital Västerås, Västerås, Sweden; 2 CHAP, Department of Public Health and Caring Sciences, Uppsala University, Uppsala, Sweden; 3 CHILD Research Environment, Jönköping University, Jönköping, Sweden; 4 Department of Mental Health, Norway Technical and Natural Sciences University, Trondheim, Norway; 5 School of Health, Care and Social Welfare, Mälardalen University, Västerås, Sweden; 6 School of Health and Welfare, Dalarna University, Falun, Sweden; 7 The Swedish School of Sport and Health Sciences, Stockholm, Sweden; Anglia Ruskin University, UNITED KINGDOM

## Abstract

**Background:**

Children with disabilities have an increased risk of mental health problems. Patterns of mental health problems and well-being may vary.

**Aims:**

To identify patterns of mental health problems and well-being in children with disabilities in Sweden, and investigate the influence of parental background (migration, education), and child cognitive level.

**Method:**

In this cross-sectional study, cluster analysis was used to analyse parents’ ratings of conduct problems, emotional symptoms, and prosocial behaviour on the Strengths and Difficulties Questionnaire (SDQ) in children with disabilities (n = 136). The influence of parental background (migration, education) and child cognitive level on cluster membership was explored through multinomial logistic regression.

**Results:**

Five clusters of mental health patterns emerged. Three clusters had mean ratings near or past clinical cut-off for one each of the SDQ-subscales. One cluster had difficulties on all three subscales. Greater child cognitive difficulties increased the likelihood of low prosocial behaviour (OR 2.501, p < .001) and of difficulties on all three subscales (OR 2.155, p = .006). Parental background did not influence cluster membership.

**Conclusion:**

Children with disabilities display varying mental health patterns. Awareness of the complexity of mental health patterns among children with disabilities is important. Screening and support for emotional symptoms and prosocial behaviour deficits should be considered for children with conduct problems.

## Introduction

Mental health problems and mental health can be seen as two different, but related, phenomena. Children with disabilities are known to have an increased risk of mental health problems compared to same-age peers [1–5]. Less is known about the extent to which children with disabilities and mental health problems also display behaviours related to mental health; that is to say how patterns of mental health problems and mental health coexist. This person-oriented cross-sectional study is intended to address this knowledge gap.

In recent decades, mental health has been proposed to consist of more than just the absence of mental health problems. Keyes [6, 7] has suggested a model in which mental health is seen as consisting of two related, but different, continua. One continuum focuses on mental health problems, from absence of mental health problems to diagnosed mental illness. The second focuses on mental health, from high mental health (flourishing) to low mental health (languishing). Keyes and others have shown that mental health problems in adults can coexist with different levels of mental health [7]. They, and other researchers [8], have suggested that high levels of mental health may protect from mental health problems.

Mental health problems in young children and children with disabilities often present as externalising and/or internalising behaviour. Therefore, mental health problems in children have frequently been operationally defined through behaviour rating scales. Rating scales of behaviour problems, however, often include items that could be more related to a child’s disability than to mental health issues. Hyperactivity, for example, is a common coexisting problem for many children with disabilities, and some may exhibit hyperactive behaviour without having conduct or emotional problems [9]. In adolescents with neurodevelopmental disorders (NDD), hyperactivity seems to be more stable over time than conduct and emotional problems, and rather be related to the NDD [4]. Likewise, peer problems may be a consequence of difficulties inherent to a child’s disability, such as communication problems. Peer problems may also be related to the behaviour of others, rather than an expression of mental health problems of the child [4, 10]. Including hyperactivity and peer problems in studies of children with disabilities may therefore lead to an overestimation of the prevalence of mental health problems. Therefore, Danielsson et al. [11] and Granlund et al. [8] recommend not using measures of hyperactivity or peer problems when investigating mental health problems in children with severe disabilities.

In Keyes two continua model [6], mental health is defined as emotional, psychological, and social well-being. Well-being measures are self-rated, making them difficult for children who are not able to respond to self-rating instruments. High levels of prosocial behaviour might be an indirect indicator of social well-being, and is reported to be related to well-being in older children [12]. Children displaying high levels of prosocial behaviour are more often positively received by others, which may contribute to strengthening further prosocial behaviour [13]. Thus, high levels of prosocial behaviour may be a protective factor against mental health problems. Children who display low levels of prosocial behaviour may on the other hand be received less well, increasing negative outcomes [8, 14].

Previous research has suggested that patterns of behavioural problems and participation in everyday situations, are more closely related to children’s level of functioning than to specific diagnoses [15]. In children with disabilities, better communication skills, adaptive behaviour and independence in everyday activities, have been found to be related to higher levels of prosocial behaviour [10]. Some studies have found greater severity of intellectual disability (ID) to be associated with higher levels of emotional and behavioural problems in children with disabilities [16, 17], while other studies [5] have found no such association.

Several factors, other than the disability itself, may contribute to patterns of mental health problems and participation in everyday situations. One of these factors may be migration background. There has been a marked increase in immigration to Sweden in recent years, with 20% of the population born outside Sweden at the end of 2021 [18]. Thus, children with disabilities with an immigrant background could constitute a substantial part of the total number of children with disabilities in Sweden. An immigrant background has in some studies been associated with a higher risk of mental health problems among children and adolescents [19, 20]. Other studies have described more varied results [21, 22]. Studies of mental health problems among children with disabilities with an immigrant background are sparse. In their study of challenging behaviour in children with ID, Dworschak, Ratz and Wagner [23] found no association with migration background.

Another factor that may have an impact on mental health patterns and participation is parental level of education. A lower level of parental education is associated with an increased risk of mental health problems [20, 24], particularly behavioural problems, in children [25, 26].

Understanding patterns of mental health problems and well-being, and factors relating to these in children with disabilities, is important for the development and targeting of support. Using a person-oriented approach, this study aims firstly to investigate whether homogeneous patterns of conduct problems, emotional symptoms and prosocial behaviour can be identified in a sample of children with disabilities in Sweden; secondly, to see if such patterns relate to parental background (immigrant/Swedish-born, level of education), and to the children’s cognitive level. In this study, children with disabilities refers to children with congenital or early onset developmental disabilities, such as ID, autism, or mobility impairments. An immigrant background refers to families where the responding parent was born in a country other than Sweden.

## Method

This is a cross-sectional study, using cluster analysis. Cluster analysis is a person-oriented method. Individuals with similar patterns of variable outcomes are grouped into clusters, allowing characteristics of individuals within clusters to be explored. This study used data from the longitudinal research programme CHILD-PMH led by the CHILD research group at Jönköping University, Sweden, and has approval from the Swedish Ethical Review Authority (reference number 2019–05028 and 2020–04810). In CHILD-PMH, data about the children’s mental health and participation, and the families’ contacts with habilitation services, are collected annually for four years from children with disabilities and their parents. The current cross-sectional study used demographic data, the Ten Question Screen (TQS), and the Strengths and Difficulties Questionnaire (SDQ) from the parent questionnaire of CHILD-PMH’s first wave of data collection (December 2020–April 2021).

### Setting and participants

**Fig 1:**
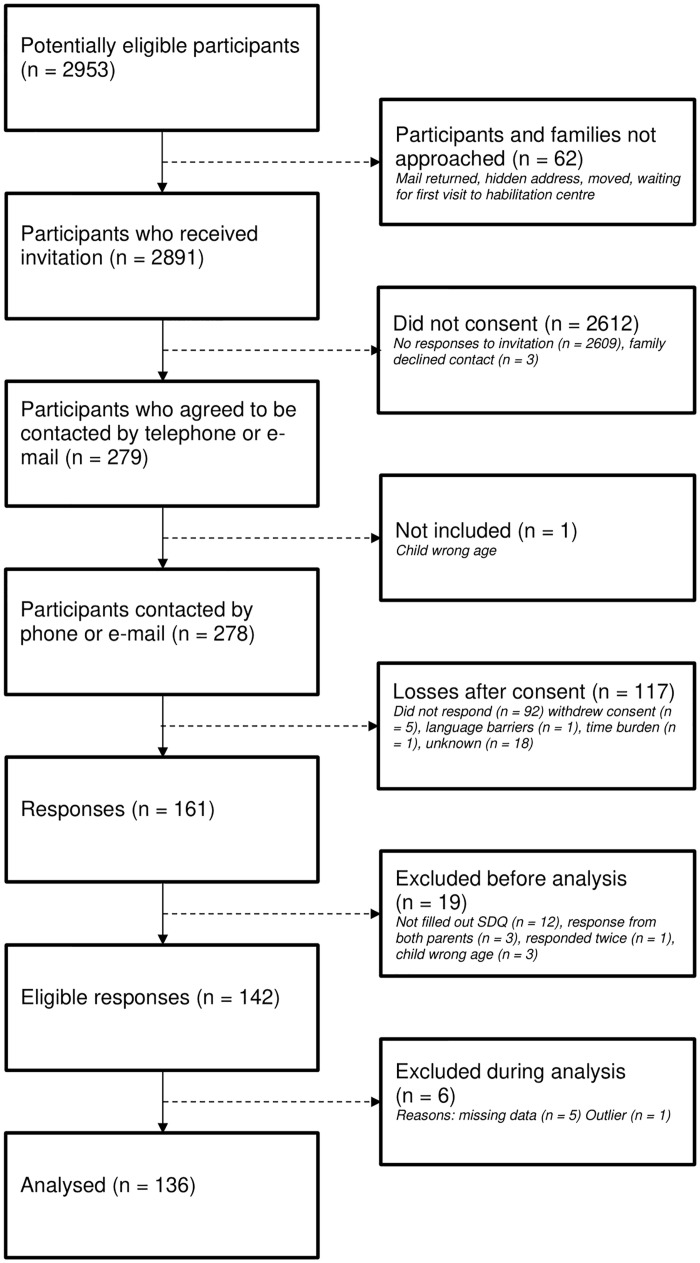
Flowchart of data collection.

In Sweden, all children with disabilities and their parents/caregivers (hereafter parents) are offered support and interventions from multidisciplinary out-patient habilitation centres. Parents of all children born in 2013–2015 (5–8 years old) and 2007–2009 (11–14 years old) (N = 2891) receiving support from the habilitation centres in five healthcare regions of Sweden were invited to take part in the CHILD-PMH programme (Fig 1). The two age cohorts were selected for the purpose of the larger longitudinal project. During the four-year period the younger cohort will grow to 9–12 years old, and the older cohort to 15–18 years old, thus providing data from children at all ages from 5–18 years during the course of the longitudinal project. However, the current cross-sectional study presented in this paper used data from the first year of data collection only. The healthcare regions were selected based on geographical convenience.

Written information about the research programme, an invitation to participate, a consent form and a prepaid return envelope were sent to the families’ homes in spring 2020. Complete written information was available in Swedish, English, Arabic and Somali (because the habilitation centres most frequently engaged interpretation services in Arabic and Somali). In autumn 2020, a reminder (in all four languages) was sent to those who had not returned the consent form.

Data collection was initially postponed due to the Covid-19 pandemic. In late autumn 2020, all families who had consented to participate by filling in and returning the written consent form (n = 278; 9.6%) were contacted by telephone and/or email to confirm their ongoing interest. Parents could choose the language of the questionnaire to respond to and whether they wanted it via email with a link to an online version, or by post with a prepaid return envelope. Reminders were sent to parents who did not reply within three weeks. Failure to respond thereafter was regarded as withdrawal from the first wave of data collection. A total of 161 parents responded.

After data collection, 19 responses were excluded from this study. Of these, 10 had answered only demographic questions, and two had not answered the SDQ. In three cases, both parents had each completed a questionnaire for the same child (only the fathers’ responses were included, to increase the father-mother ratio of respondents). One questionnaire had been filled out twice by the same parent for the same child, and three children were not born in the specified years. These exclusions resulted in a total of 142 cases.

### Instruments

#### Ten Question Screen (TQS)

In the CHILD-PMH programme, the Ten Question Screen (TQS), rather than the children’s diagnoses, was used as indicator of the children’s level of functioning. The TQS was originally developed to screen for cognitive delay and other disabilities in children in diverse cultures [27]. It comprises 10 questions regarding the child’s level of understanding (cognitive ability), learning, communication, and motor skills, as well as difficulties with sight, hearing, or seizures. For the purposes of the CHILD-PMH programme, the following question was added (10a): ‘How would you describe the level of difficulty your child has in understanding?’, with ‘mild’, ‘moderate’, ‘severe’ and ‘very severe’ as possible answers. In the present study, the answers to the TQS question number 10 (‘compared to other children of the same age, does your child seem to have difficulties understanding or appear mentally slow?’) indicating cognitive delay [28], and the answers to the added question 10a, were combined, giving following five levels: ‘no difficulties’ (those who answered ‘no’ to question 10), ‘mild’, ‘moderate’, ‘severe’ and ‘very severe’. This was used as a proxy rating of the child’s cognitive level.

#### Strengths and Difficulties Questionnaire (SDQ)

The SDQ [29] is commonly used worldwide to screen for emotional and behavioural issues in children. It exists in parent, teacher, and self-report (from 11 years) versions. In this study, the parent version was used, which includes twenty-five items rated as ‘not true’, ‘somewhat true’ or ‘certainly true’ on conduct problems, hyperactivity, emotional symptoms, peer problems and prosocial behaviours subscales. The results of the four problem subscales (not the prosocial scale) can be combined into a total difficulties score. For children with disabilities this may be problematic, however [8, 11]. As hyperactivity and peer problems could reflect the child’s disability rather than mental health problems, only the parents’ ratings on the conduct problems, emotional symptoms and prosocial behaviour subscales were included.

While there are Swedish norms for preschool children [30], children aged 6–10 [31], and 10–13 year olds [32], combined Swedish norms for the whole age range of this study are lacking. The United Kingdom cut-off values for parent-rated SDQs for ages 4–17 [33] were therefore used. Previous research [34] has found the parent-rated SDQ to be a useful measure of behavioural and emotional problems in children with intellectual disabilities, and supports the use of the same SDQ cut-offs for these children.

### Analysis

Authors had access to coded data only. Statistical analysis was performed using ROPstat [35] and IBM SPSS version 26. Following Vargha, Torma & Bergman [35], a residual analysis was conducted before the cluster analyses. The default threshold of an average squared Euclidian distance of .7 from the nearest neighbour was used. Ward’s method of agglomerative hierarchical cluster analysis was used to find a suitable number of clusters. The criteria were that the solution should have an explained error sum of squares (EESS) percentage around 67%, have homogeneity coefficients below 1, preferably consist of no more than 15 and no less than five clusters and be theoretically relevant [36]. K-means cluster analyses were then performed to compare and improve solutions by the relocation of cases.

Multinomial logistic regression was then used to investigate whether cluster membership was related to parental background (immigrant/Swedish-born, educational level), or to the child’s cognitive level. Cluster membership was treated as the dependent variable, parent immigrant/Swedish-born and parent educational level as factors and child’s cognitive level as a covariate. Whether child gender influenced the clusters was examined with Pearson’s chi-square test.

## Results

### Characteristics of the participants

Characteristics of the children and parents in the study are presented in Tables 1 and 2. Most parents (90%, n = 123) filled out the Swedish version of the questionnaire. Five parents (4%) chose the English and eight (6%) the Arabic version. The Somali version was not used by any parent. If the parent who completed the questionnaire was born in a country other than Sweden, they were counted as immigrant for the purpose of this study (44%). Seven children had one Swedish-born and one foreign-born parent. In six of these cases the respondent was the Swedish-born parent and was therefore not counted as immigrant. In the seventh case the parents had filled out the questionnaire together using the Arabic version and were therefore counted as immigrant. Responding immigrant parents (n = 60) were born in 21 different countries and had lived in Sweden between 1 and 41 years ($\bar{x}$ 10.31 years, s 7.73 years; missing: 3%, n = 2).

**Table 1:** Characteristics of the children included in this study, by age cohort and in the total sample.

Characteristic	5–8 years		11–14 years		Total	
	*n*	(%)	*n*	(%)	*n*	(%)
Gender						
Girl	21	(30)	24	(36)	45	(33)
Boy	48	(70)	42	(63)	90	(66)
Other	0	(0)	1	(1)	1	(1)
Cognitive level						
No difficulties	19	(28)	23	(34)	42	(31)
Mild	9	(13)	20	(30)	29	(21)
Moderate	21	(30)	10	(15)	31	(23)
Severe	12	(17)	7	(10)	19	(14)
Very severe	8	(12)	5	(7)	13	(10)
Missing	0	(0)	2	(3)	2	(1)
Age (*M*, *SD*)	6.54	0.82	12.56	0.87	9.50	3.13

*Note*. N = 136 (n = 69 for 5–8-year-olds, n = 67 for 11-14-year-olds).

**Table 2:** Sociodemographic characteristics of responding parents included in this study, by child age cohort and in the total sample.

Characteristic	5–8-years		11–14-years		Total	
	*n*	(%)	*n*	(%)	*n*	(%)
Gender						
Male	11	(16)	9	(13)	20	(15)
Female	27	(39)	30	(45)	57	(42)
Completed by both parents together	20	(29)	20	(30)	40	(29)
Missing	11	(16)	8	(12)	19	(14)
Education						
< 9-year/other	2	(3)	5	(7)	7	(5)
9-year elementary school	5	(7)	7	(10)	12	(9)
Upper secondary school	23	(33)	24	(36)	47	(35)
University	34	(49)	31	(46)	65	(48)
Missing	5	(7)	0	(0)	5	(4)
Place of birth						
Sweden	34	(49)	42	(63)	76	(56)
Europe	4	(6)	3	(4)	7	(5)
Middle East/S.Asia	18	(26)	15	(22)	33	(24)
Africa	12	(17)	6	(9)	18	(13)
S. America	0	(0)	1	(1)	1	(1)
Missing	1	(1)	0	(0)	1	(1)
Immigrant time in Sweden, years (*M*, *SD*)	8.36	4.51	12.88	10.13	10.31	7.73

*Note*. N = 136 (n = 69 for 5–8-year-olds, n = 67 for 11-14-year-olds).

Parents were asked to rate their level of education on a four-graded scale, from nine-year elementary school to university (Table 2). In Sweden, nine-year elementary school is compulsory, while higher levels of education are optional. In the total sample, 5% (n = 7) had not used the scale but instead written an answer indicating less than nine-year elementary school, either specifying a shorter education (for example “illiterate”, “no education” or “five-year school”) or only writing “SFI” (Swedish for immigrants). Their level of education was classified as “less than nine-year elementary school/other”.

### Initial analyses

The residual analysis identified one outlier. This case and five cases with missing SDQ-data were excluded from the cluster analyses, leaving a sample size of 136 (Fig 1). Spearman’s rank correlation revealed a weak positive correlation between a) respondent immigrant/Swedish-born and c) the child’s cognitive level (r = .23, p = .009). A weak negative correlation occurred between a) respondent immigrant/Swedish-born and b) respondent’s educational level (r = -.23, p = .008). No significant correlation appeared between a) respondent’s educational level and c) the child’s cognitive level (r = -.10, p = .239). Thus, multicollinearity was ruled out, enabling the use of multinomial logistic regression.

### Patterns of conduct problems, emotional symptoms, and prosocial behaviour

A five-cluster solution was chosen according to the specified criteria. This solution accounted for 67.62% EESS and had a mean homogeneity coefficient (HC) of 0.669 and a HC range of 0.45–1.10, with all but one cluster with HC <1 (Table 3). Mean scores for each cluster were compared to the cut-off value for the “high” band of each subscale in the four-band UK-scoring of the parent rated SDQ for 4-17-year olds: ≥4 for conduct problems, ≥5 for emotional symptoms and ≤6 for prosocial behaviour [33] (Table 3).

**Table 3:** Profiles of SDQ subscale scores, homogeneity coefficients and cluster sizes for each cluster.

Cluster	Conduct problems		Emotional symptoms		Prosocial behaviour		HC	Size
	M	SD	M	SD	M	SD		
CL1	0.73	0.84	1.42	1.17	8.06	1.52	0.45	33
CL2	1.80	1.04	**6.56**	1.53	7.76	1.74	0.66	25
CL3	2.03	1.20	2.16	1.64	**2.68**	1.62	0.72	31
CL4	3.86	1.04	2.61	1.45	6.57	1.57	0.59	28
CL5	**5.95**	1.62	**6.32**	2.08	**3.37**	1.74	1.10	19

*Note*. Values past cut-off for the high band of the UK four-band scoring for parent rated SDQ 4–17 years are shown in bold. Cut off values are ≥ 4 for conduct problems, ≥ 5 for emotional symptoms ≤ 6 for prosocial behaviour.

**Fig 2:**
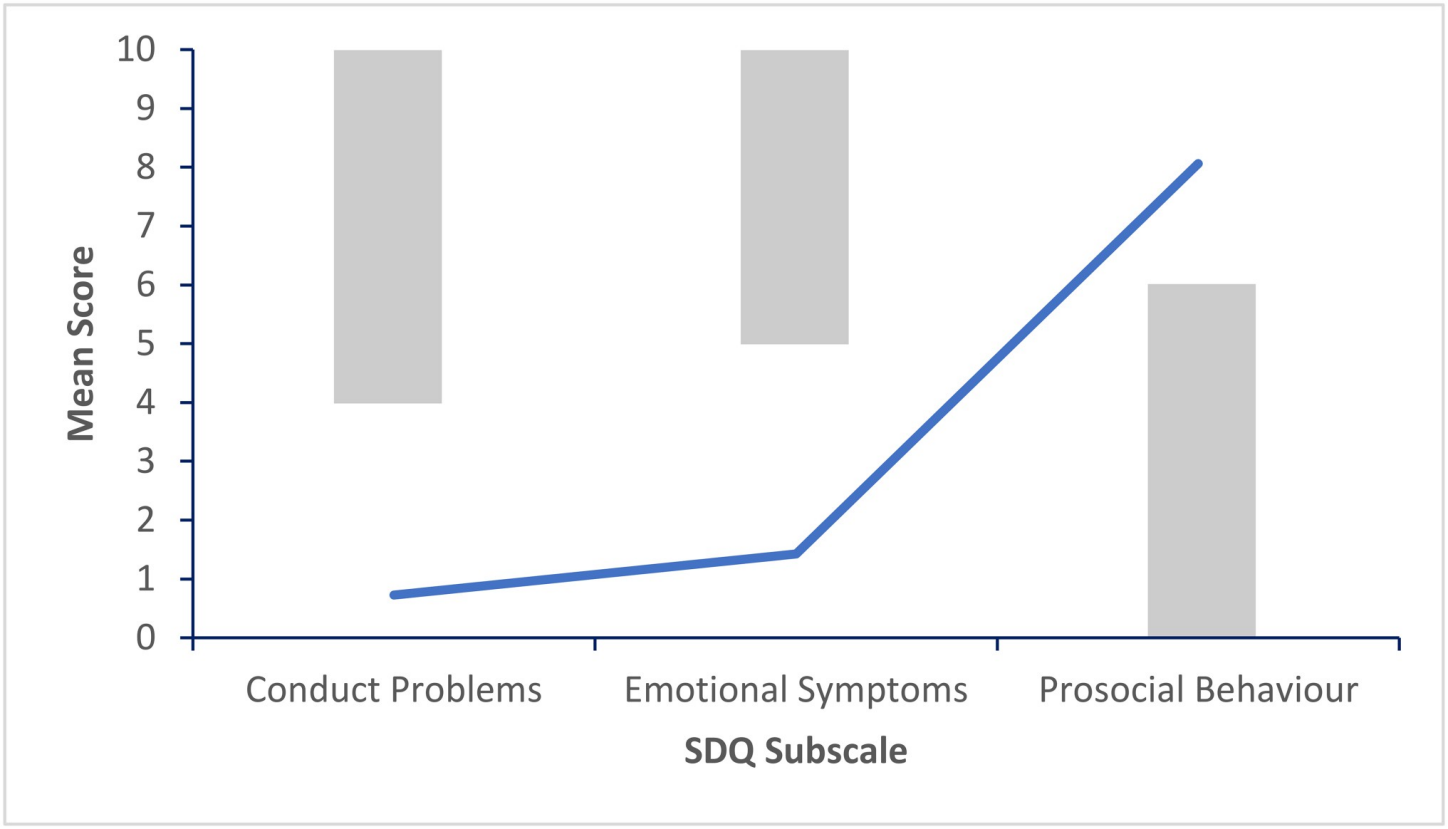
CL1 profile of SDQ scores on the subscales conduct problems, emotional symptoms and prosocial behaviour. *Note*. The shaded areas show the combined high and very high bands of the UK four-band scoring for parent rated SDQ 4–17 years.

**Fig 3:**
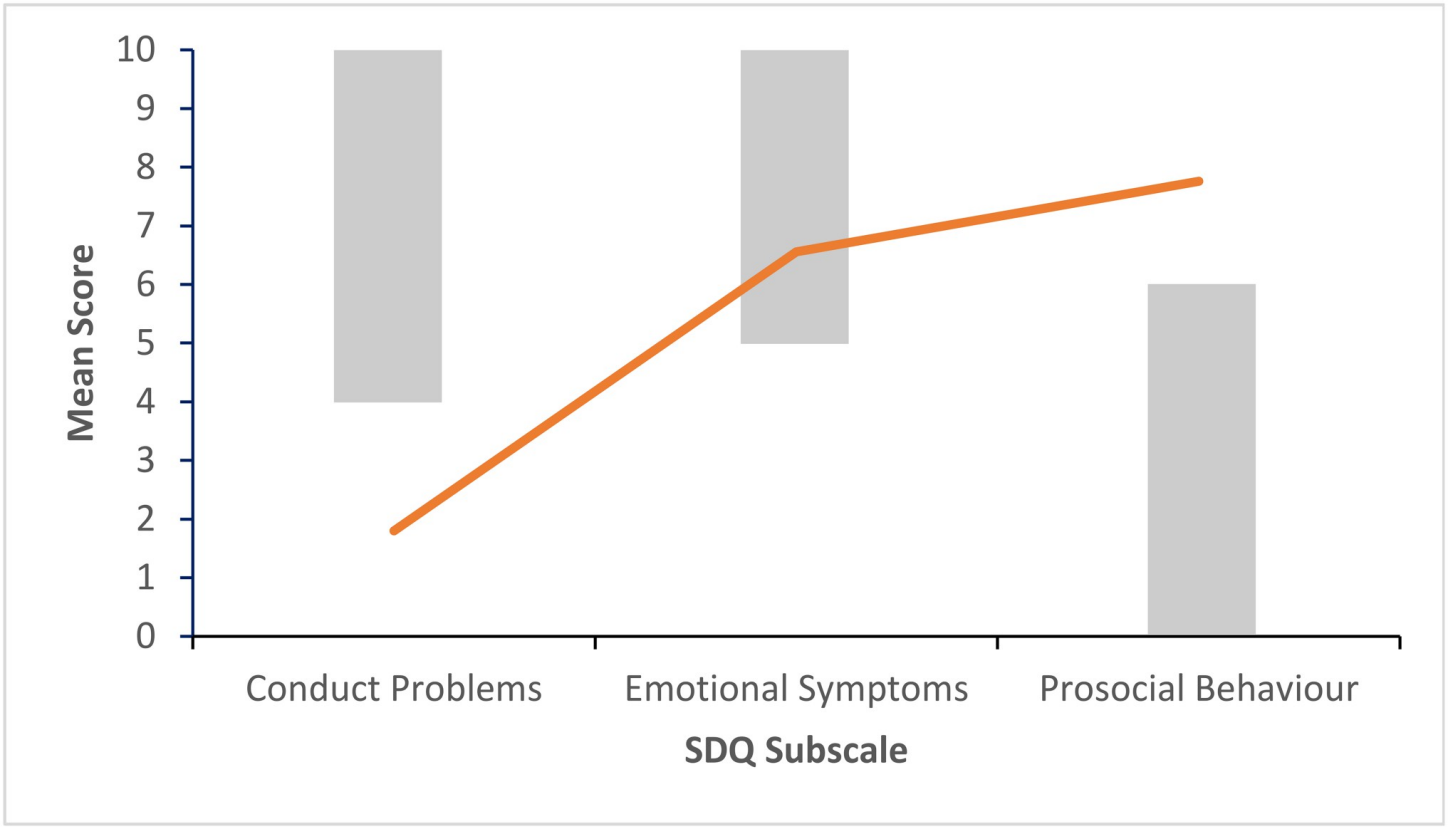
CL2 profile of SDQ scores on the subscales conduct problems, emotional symptoms and prosocial behaviour. *Note*. The shaded areas show the combined high and very high bands of the UK four-band scoring for parent rated SDQ 4–17 years.

**Fig 4:**
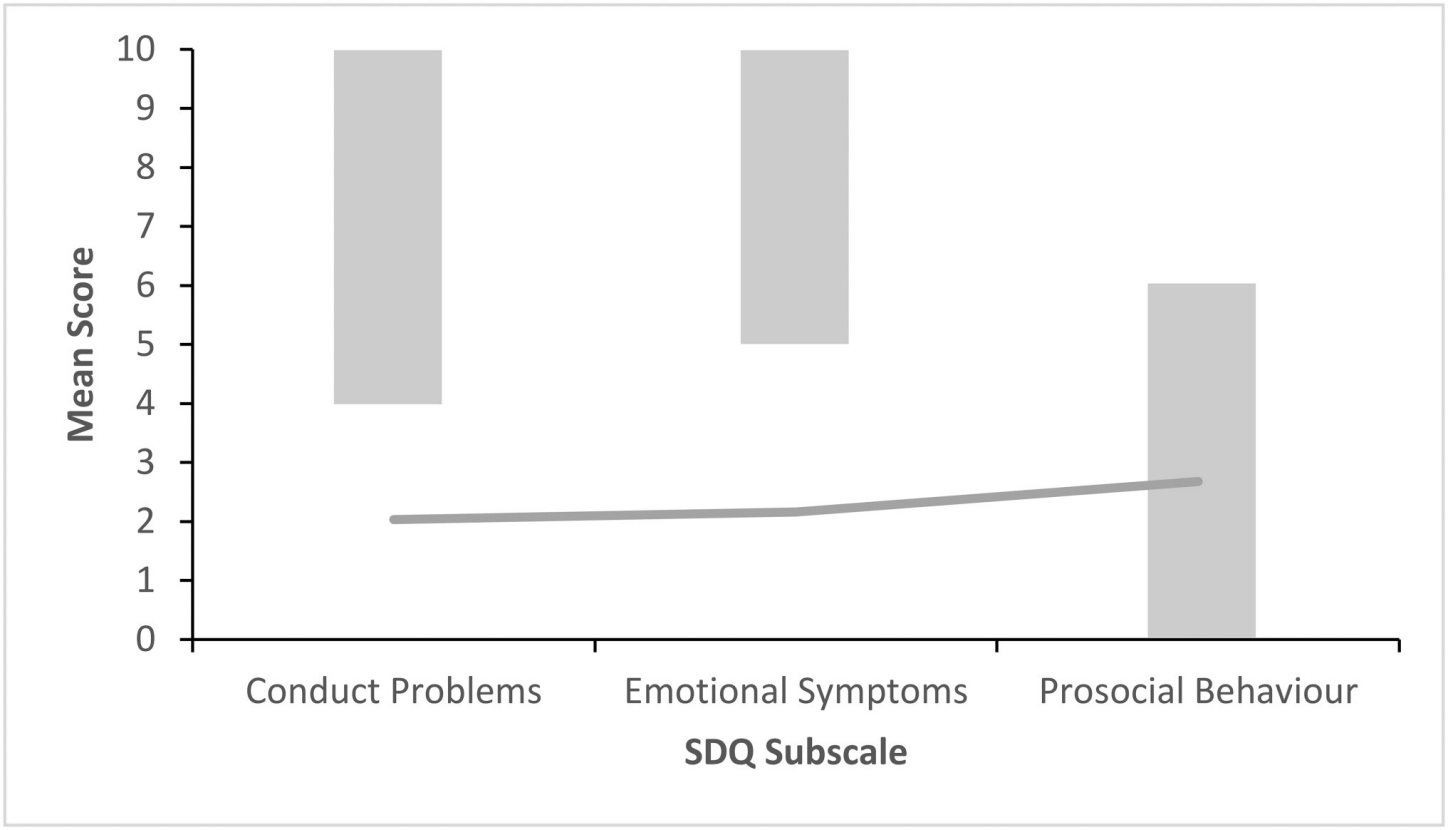
CL3 profile of SDQ scores on the subscales conduct problems, emotional symptoms and prosocial behaviour. *Note*. The shaded areas show the combined high and very high bands of the UK four-band scoring for parent rated SDQ 4–17 years.

**Fig 5:**
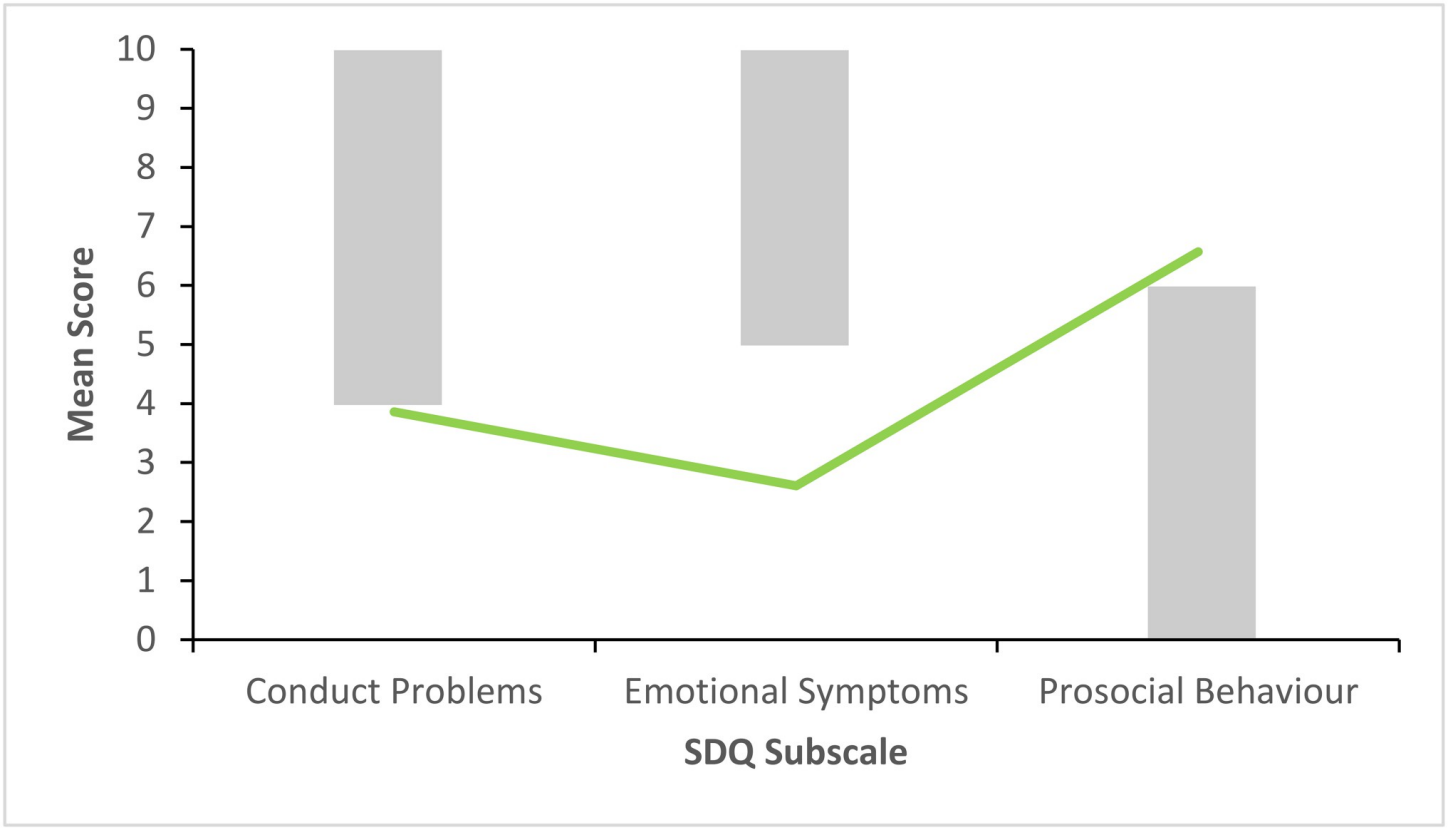
CL4 profile of SDQ scores on the subscales conduct problems, emotional symptoms and prosocial behaviour. *Note*. The shaded areas show the combined high and very high bands of the UK four-band scoring for parent rated SDQ 4–17 years.

**Fig 6:**
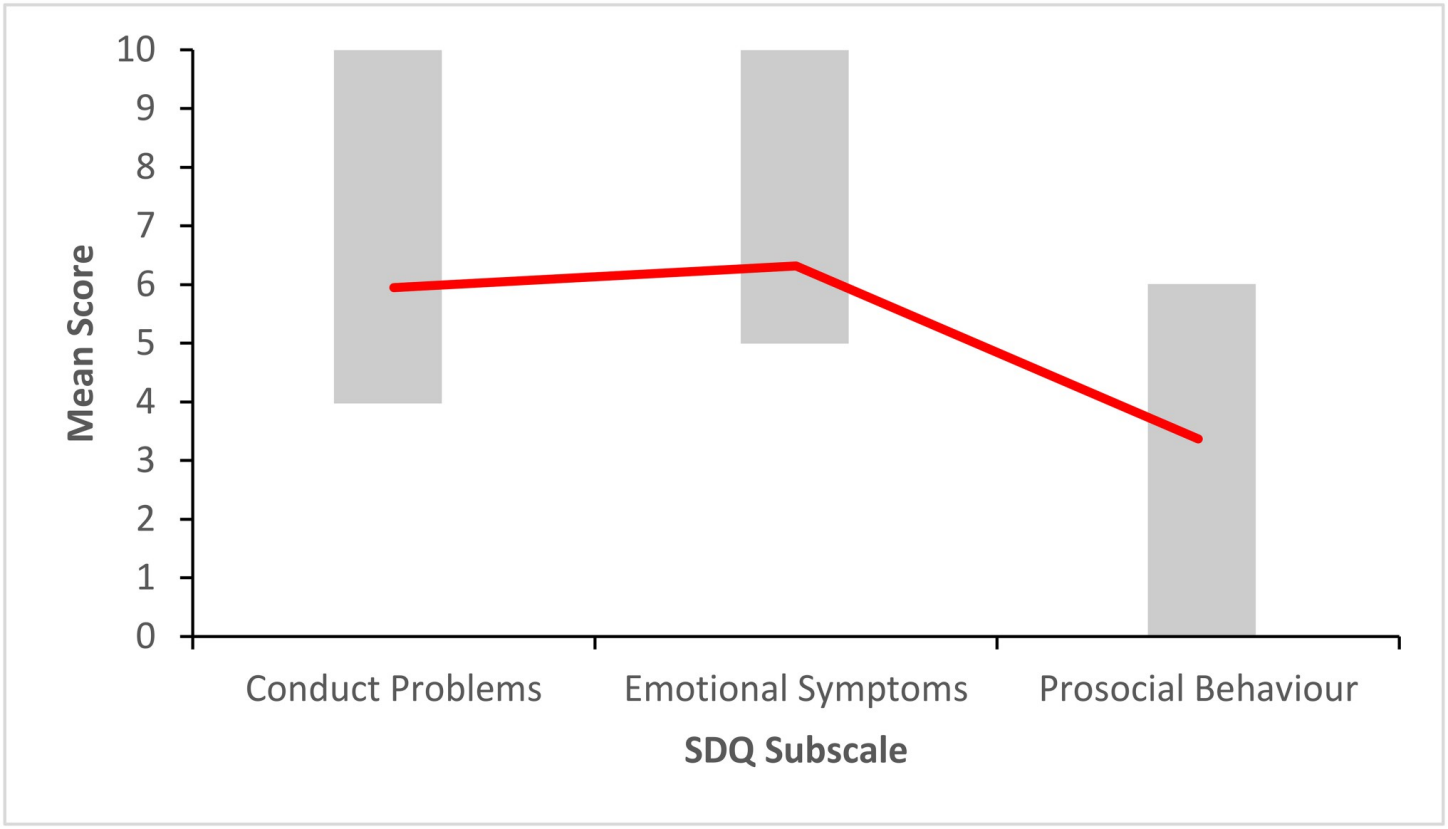
CL5 profile of SDQ scores on the subscales conduct problems, emotional symptoms and prosocial behaviour. *Note*. The shaded areas show the combined high and very high bands of the UK four-band scoring for parent rated SDQ 4–17 years.

Figs 2–6 show the pattern of mean subscale scores per cluster. The shaded areas show the combined high and very high bands of the UK four-band scoring for parent rated SDQ 4–17 years.

None of the mean scores on the three subscales in CL1 (Fig 2) indicated difficulties. Clusters 2 to 4 (Figs 3–5) had mean scores bordering on or past the cut-off levels for problems on one each of the three subscales. CL2 displayed a pattern of primarily emotional symptoms, CL3 prosocial difficulties, and CL4 conduct problems. CL5 (Fig 6) had mean ratings past the cut-off values for problems on all three subscales.

### Clusters in relation to parental background and child cognitive level

Table 4 shows a descriptive summary of the clusters.

**Table 4:** Characteristics of the children in each cluster.

Characteristic	CL1		CL2		CL3		CL4		CL5	
	*n*	(%)	*n*	(%)	*n*	(%)	*n*	(%)	*n*	(%)
Child age										
5–8 years	12	(36)	9	(36)	20	(65)	19	(68)	9	(47)
11–14 years	21	(64)	16	(64)	11	(35)	9	(32)	10	(53)
Child gender										
Boys	20	(61)	16	(64)	22	(71)	21	(75)	11	(58)
Girls	13	(39)	8	(32)	9	(29)	7	(25)	8	(42)
Other	0	(0)	1	(4)	0	(0)	0	(0)	0	(0)
Child cognitive level										
No difficulties	13	(39)	8	(32)	6	(19)	9	(32)	6	(32)
Mild	11	(33)	7	(28)	4	(13)	5	(18)	2	(11)
Moderate	6	(18)	7	(28)	6	(19)	7	(25)	5	(26)
Severe	2	(6)	1	(4)	10	(32)	6	(21)	0	(0)
Very severe	0	(0)	2	(8)	5	(16)	1	(4)	5	(26)
Missing	1	(3)	0	(0)	0	(0)	0	(0)	1	(5)
Parent place of birth										
Sweden	19	(58)	15	(60)	21	(68)	14	(50)	7	(37)
Outside Sweden	14	(42)	10	(40)	10	(32)	14	(50)	12	(63)
Parent education										
< 9year/other	4	(12)	1	(4)	1	(3)	1	(4)	0	(0)
9 year elementary	4	(12)	0	(0)	2	(6)	2	(7)	4	(21)
Upper secondary	9	(27)	9	(36)	14	(45)	9	(32)	6	(32)
University	16	(49)	15	(60)	14	(45)	13	(46)	7	(37)
Missing	0	(0)	0	(0)	0	(0)	3	(11)	2	(11)

*Note*. N = 136 (CL1 n = 33, CL2 n = 25, CL3 n = 31, CL4 n = 28, CL5 n = 19)

CL1 was the largest cluster. In this cluster the SDQ ratings showed no conduct, emotional or prosocial difficulties, and the children had the least cognitive difficulties of all clusters. This cluster had a few more Swedish born than immigrant parents and half of the parents had a university education.

In CL2, SDQ scores primarily indicated emotional symptoms. Most of the children had none to moderate cognitive difficulties. More than half of the parents were Swedish born, and the same proportion had a university education.

CL3 primarily had prosocial difficulties, and the largest number of children was found in the ‘severe cognitive difficulties’ category. Remaining children were fairly equally spread between the other four cognitive levels. In this cluster two thirds of parents were born in Sweden. There was an equal number of parents with a university and an upper secondary school education.

CL4, with a mean rating bordering on the cut-off level for conduct problems, had the largest number of children in the ‘no cognitive difficulties’ category. This cluster had an equal number of Swedish born and immigrant parents, almost half with a university education.

CL5, the smallest cluster, had mean scores indicating difficulties on all three SDQ subscales. There was a similar proportion of children with no cognitive difficulties and children with very severe cognitive difficulties in this cluster. This was the only cluster with fewer Swedish born than immigrant parents. An almost equal proportion of parents had a university and an upper secondary school education.

Multinomial logistic regression, with cluster membership as the dependent variable and CL1 as the reference category, showed that the probability of a child being in CL3 or CL5 was affected by child cognitive level. It was more likely that a child with cognitive difficulties would be in CL3 (OR 2.501, 95% CI 1.535–4.074, p < .001) or CL5 (OR 2.155, 95% CI 1.250–3.717, p = .006) than in CL1. High level of cognitive difficulties increased the likelihood of the child having a pattern of primarily prosocial difficulties or difficulties on all three SDQ subscales. Neither responding parent being Swedish-born or immigrant, nor parental level of education, affected the child’s cluster membership. Pearson’s chi-square test showed that child gender did not influence the clusters (p = .555).

## Discussion

The aim of this study was firstly to investigate whether patterns of mental health problems (conduct problems and emotional symptoms) and well-being (prosocial behaviour) could be identified in a sample of children with disabilities; secondly, to see if such patterns were related to background factors in terms of parental immigration or not, parental level of education, and the child’s cognitive level.

### Patterns of mental health problems and well-being

As expected, the children in this study displayed varying patterns of mental health problems and well-being, and could be grouped together based on these patterns. The sample was too small to allow for statistical analysis of the differences between clusters, but differences can still be discussed. Some children, in CL5, experienced difficulties in all three areas (conduct problems, emotional symptoms and prosocial behaviour), indicating both a presence of mental health problems and a lack of well-being. It is important to note that parents of children with high levels of conduct problems are likely to seek support from health care services [37]. This is because aggressive, oppositional, or defiant behaviour can be disruptive and directly affect other people. Emotional symptoms, however, are often less disruptive and could be missed. Other children, in CL4 and CL2, displayed patterns of simultaneous mental health problems and well-being, in accordance with the two continua model [7]. These children may have an advantage in their prosocial skills as a protective factor against mental health problems [8].

One group of children, in CL3, had low levels of prosocial behaviour without conduct problems or emotional symptoms. These children appear not to have mental health problems, but could at the same time be lacking in well-being through their deficits in prosocial behaviour [8].

Differing patterns of mental health problems and well-being in children with disabilities have clinical implications. When supporting children with disabilities who have conduct problems and their parents, an awareness of possible coexisting emotional symptoms and prosocial difficulties is also important. As seen in CL5, some children with disabilities have both conduct problems, emotional symptoms, and a lack of prosocial behaviour. Conduct problems could mask emotional symptoms and prosocial deficits. Support should incorporate not only interventions aimed at reducing conduct problems, but also at addressing emotional symptoms and enhancing prosocial skills, to increase the child’s resilience against mental health problems. Children with primarily conduct problems, who at the same time display high levels of prosocial behaviour and low levels of emotional symptoms (CL4), also need support, despite the possible protective presence of high prosocial skills [8]. Support is important to minimise the immediate problems, as well as to avoid increased conduct problems over time [9, 14], and reduce the risk of developing emotional problems, as conduct problems in childhood carry an increased risk of depression in adulthood [38].

Children without conduct problems, but with emotional symptoms (CL2) or prosocial deficits (CL3) may not cause problems for others and may thus receive less attention and/or support. This has been demonstrated previously, for example in a study by Almqvist et al. [39]. They investigated predictors of young children receiving support in preschool and found that low engagement in preschool activities but no exhibited behaviour problems, resulted in very low likelihood of receiving support. Absence of mental health problems but simultaneous lack of well-being through low levels of prosocial behaviour (CL3) may also be problematic. Rai et al. [40] found deficit in social communication in childhood to be associated with depression in young adulthood. Interventions to increase communication and social skills in these children could be important also from a mental health perspective. Thus, a clinical awareness of the existence of these mental health patterns among children with disabilities is important so that children who may need support for mental health issues can be identified.

While this study found various patterns of mental health problems and well-being in children with disabilities, the largest cluster of children in this study (CL1) had no apparent mental health problems and had high levels of well-being.

### Parental background and children’s cognitive level

Parental background did not influence child pattern of mental health in this sample. Previous research has presented varied results regarding whether an immigrant background may be a potential risk factor for mental health problems in children [21, 22]. The largest group of children in this study (CL1) did not appear to have mental health problems. In this group, 42% of parents were born outside Sweden. Some factors discussed by Belhadj Koudier, Koglin & Petermann [21], often associated with migration, for example socioeconomic status, family situation and discrimination, may be risk factors for children’s mental health. These factors, independent of migration, could also be experienced by families of children with disabilities. It may be that possible effects of stressors related to an immigrant background are overshadowed by consequences of stressors related to the child’s disability. It may also be that the sample was too small and heterogenous for effects of an immigrant background to show. Immigrant parents in this study had a very wide range of backgrounds in terms of country of origin and time in Sweden. They were not asked about their immigration status, and experiences can be presumed to differ depending on place of origin and reason for migration [41]. A more homogenous or larger sample enabling subgroup analyses would be needed to further explore if an immigrant background is related to patterns of mental health in children with disabilities.

The effect of parental education level on the mental health of children with disabilities is understudied. This study, like that of Brossard-Racine et al. [10], did not find an association between parents’ educational level and SDQ-scores. However, most parents in this study had at least upper secondary schooling, half had a university education. Education and income are often used as indicators of socioeconomic status. In this study data on family income was not available. Education alone is not necessarily equal to socioeconomic status, particularly among recently arrived immigrants. A measure of family income together with parental education may have been more relevant.

The child’s cognitive level did, however, influence mental health patterns. Greater cognitive difficulties increased the likelihood of prosocial difficulties or difficulties with conduct problems, emotional symptoms, and prosocial behaviour. This is in line with previous research that has found an association between greater severity of intellectual disability and higher levels of mental health problems [1, 16, 17], and that children with higher levels of cognitive ability also had higher levels of prosocial behaviour [10]. Contradicting this, Buckley et al. [5] did not find differences in psychiatric symptoms according to level of ID in their meta-analysis.

### Strengths and limitations

A strength of this study is the use of person-oriented analysis. A person-oriented approach is based on the holistic view that each individual has their own specific combination of properties. In this study, the person-oriented cluster analysis provided a nuanced view of mental health among children with disabilities, as subgroups of children with similar patterns of covarying mental health problems and well-being emerged. A traditional variable-oriented analysis would rather have provided a group-based view where mental health problems have a negative relationship with prosocial behaviour [42, 43], a result that is only true for some children in the sample. Much of previous research into the mental health of children with disabilities has primarily been variable-oriented. An increased awareness that children with disabilities can display varying patterns of mental health problems and simultaneous lack or presence of well-being could facilitate more individually tailored support.

The study contains some important limitations. Firstly, a low response rate and an unknown population representativity means results must be interpreted with caution. Also, all data is parent-reported, entailing a risk of bias.

UK, rather than Swedish, cut-off points for the SDQ were used. These are, however, very similar to the Swedish norms for children in the 6–13 age range [31, 32]. As reasoned by Parkes et al. [1], some items of the SDQ may be less relevant for children with for example severe mobility impairment, who may not be able to carry out described behaviours. Other researchers, e.g. Downs et al. [3], argue that some aspects measured with the SDQ may rather reflect neurocognitive delay or physical or communication difficulties than mental health problems. In this study the hyperactivity subscale and the peer problems subscale were not part of the analysis, for similar reasons. It may be that even the subscales used are not optimal for screening for mental health problems in children with some disabilities. Furthermore, Belhadj Koudier, Koglin & Petermann [21] warn that parents with another ethnic background may not necessarily understand the items of the SDQ correctly, due to language difficulties or a different understanding of mental health. In this study a few parents used the English or Arabic versions of the SDQ, no parent used the Somali version. It is possible that remaining parents felt comfortable using the SDQ in Swedish, but it could also be that some used the Swedish version for lack of a more suitable one.

Social well-being is an important part of well-being, and it can be argued that prosocial behaviour can be seen as an indicator of this. Yet, the use of the prosocial behaviour subscale of the SDQ must be interpreted with caution. Well-being is of course a far broader concept than prosocial behaviour alone. It may be that a different rating scale of well-being would have given other results. There is, however, a lack of instruments to measure well-being in children, particularly for children not able to respond to self-rating scales.

The TQS question about understanding (question 10) together with the added question about the level of difficulty were used as an approximation of the child’s cognitive level in this study. This gives a rough estimate, but it is of course not certain that the parents’ ratings equate to the child’s actual intellectual level.

The heterogeneity regarding background factors, especially time spent in Sweden, did not allow for analyses of potential mediating factors. Lack of information regarding immigrant parents’ reasons for migrating to Sweden is also a limitation, as is lack of information about parents’ own health which also could contribute to children’s mental health problems. The results concerning the relation between background factors and patterns of mental health problems should thus also be interpreted with care.

The study was postponed due to Covid-19. During the Covid-19 pandemic Swedish schools and kindergartens remained open and there were no enforced quarantines or ‘lock-downs’ [44]. The mental health of the children in this study may still have been affected by the pandemic, but no data enabling a specific analysis of this was collected.

## Conclusion

Children with greater cognitive difficulties seem to have a higher risk of prosocial behaviour deficit or a combination of conduct problems, emotional symptoms, and low prosocial behaviour. This study did not, however, find any support for parental background influencing child mental health patterns. Conduct problems and/or emotional symptoms can be present while the child at the same time displays well-being in terms of positive social functioning through prosocial behaviour. These results support Keyes’ two continua model. From a mental health perspective, it may be important to screen for emotional symptoms and to consider the child’s level of well-being (prosocial behaviour) even in children whose parents do not seek support for this, particularly in children with cognitive difficulties.
